# *ZafA* Gene Is Important for *Trichophyton mentagrophytes* Growth and Pathogenicity

**DOI:** 10.3390/ijms20040848

**Published:** 2019-02-15

**Authors:** Pengxiu Dai, Yangou Lv, Yongping Gao, Xiaowen Gong, Yihua Zhang, Xinke Zhang

**Affiliations:** The College of Veterinary Medicine of the Northwest Agriculture and Forestry University, No. 3 Taicheng Road, Yangling 712100, China; dpx910405@163.com (P.D.); lvyangou@163.com (Y.L.); gaoyongping@163.com (Y.G.); gongxiaowen1983@sina.com (X.G.)

**Keywords:** *Trichophyton mentagrophytes*, *ZafA*, *Agrobacterium tumefaciens*-mediated transformation, growth, pathogenicity

## Abstract

*Trichophyton mentagrophytes* is a common fungal pathogen that causes human and animal dermatophytosis. Previous studies have shown that zinc deficiency inhibits *T. mentagrophytes* growth, and the *ZafA* gene of *T. mentagrophytes* can code the functionally similar zinc finger transcriptional factor that can promote zinc ion absorption; however, the impact of *ZafA* on virulence and pathogenicity remains undetermined. To assess its gene function, the *ZafA* mutant, ZafA-hph, and the *ZafA* complemented strain, ZafA+bar, were constructed via *Agrobacterium tumefaciens*-mediated transformation. Polymerase chain reaction and Southern blot analyses were used to confirm the disruption. In vitro growth capacity and virulence analyses comparing ZafA-hph with wild-type *T. mentagrophytes* and ZafA+bar showed that ZafA-hph’s growth performance, reproduction ability, and zinc ion absorption capacity were significantly lower than the wild-type *T. mentagrophytes* and ZafA+bar. ZafA-hph also showed weak hair biodegradation ability and animal pathogenicity. Thus, the significant decrease in *T. mentagrophytes’* growth ability and virulence was due to a lack of the zinc-responsive activity factor rather than the transformation process. This study confirmed that the *T. mentagrophytes’* zinc-responsive activity factor plays important roles in the pathogen’s growth, reproduction, zinc ion absorption, and virulence. This factor is important and significant for effectively preventing and controlling *T. mentagrophytes* infections.

## 1. Introduction

*Trichophyton mentagrophytes* is a common fungal pathogen worldwide that causes human and animal dermatophytosis and severe skin infections [1,2,3]. These pathogens can invade keratinized tissue such as skin, nails, and hair [4]. Many epidemiological studies have shown that *T. mentagrophytes* is globally distributed, and is one of the most frequent or common pathogens of fungal skin disease [5,6,7,8]. Some animals, including rabbits, foxes, minks, cats, and dogs are the natural hosts of *T. mentagrophytes*, which causes skin diseases in these animals and affects their fur quality [9,10]. In cases of low immunity or a damaged skin barrier, close contact with the above-mentioned sick animals can easily result in fungal skin diseases [11]. Thus, *T. mentagrophytes* is considered a public health hazard because of the high possibility for zoonosis [12,13,14].

Zinc is an important micronutrient, which is often combined with a variety of functional proteins in fungi and other microorganisms to form zinc finger proteins for biological functions. Over the life of the fungi, their cells must obtain zinc ions to grow and be pathogenic; thus, to hinder pathogenic fungal growth, mammalian hosts reduce the concentration of free zinc ions in their cells [15,16]. To maintain the appropriate zinc ion concentration and ensure normal functional operations, fungi have a complex zinc absorption transport control system [17]. Therefore, the zinc absorption transport system, which is highly expressed at relatively low zinc concentrations, is important for fungal invasion and pathogenicity [3]. Zinc absorption transport system expression in the model fungus, *Saccharomyces cerevisiae*, is primarily regulated by the C_2_H_2_-type zinc finger transcription factor, *Zap1*, at the transcriptional level [3]. Studies have shown that various fungi can code functionally similar zinc finger transcriptional factors. For example, in the fungi *Aspergillus fumigatus*, *Candida albicans*, and *Cryptococcus gattii*, mutations in similar zinc absorption transport mechanism genes can stop growth and development and even cause virulence loss [18,19,20]. Previous studies have shown that zinc deficiency can inhibit *T. mentagrophytes* growth, and the *ZafA* gene of *T. mentagrophytes* can also code the functionally similar zinc finger transcriptional factor, whose composition has been determined, but whose impact on virulence and pathogenicity is undetermined [21].

Reverse genetics is an effective means of investigating a potential virulence gene’s function by disrupting the target gene and analyzing the mutated strain [22], and loss of function has to be corroborated by the restoration of function via complementation. Among various fungal transformation techniques, *Agrobacterium tumefaciens*-mediated transformation (ATMT) has become a preferred transformation method for researchers because of its high conversion efficiency and transformant stability [23]. ATMT has been successfully applied to many fungi, including *S. cerevisiae*, *T. mentagrophytes*, and six *ascomycetes* and *basidiomycetes* species [24,25,26,27,28].

The current study evaluated the *ZafA* gene’s role in *T. mentagrophytes’* growth capacity and pathogenicity. The *ZafA* mutant, ZafA-hph, and the *ZafA* complemented strain, ZafA+bar, were constructed by ATMT for comparative studies with the wild-type *T. mentagrophytes* on growth phenotype and pathogenic changes.

## 2. Results

### 2.1. Confirmation of T. mentagrophytes’ ZafA Mutation and Restoration

**Figure 1:**
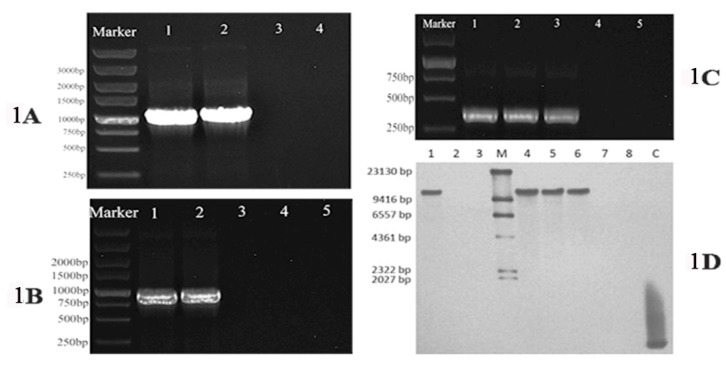
(**A**): The polymerase chain reaction results of *hph* gene (1200 bp) amplification; the inserted *hph* gene was detected with primers *hph*-F and *hph*-R. Lane 1: *pDHt/ZafA::hph*, Lane 2: ZafA-hph, Lane 3: ZafA+bar, Lane 4: The wild-type *T. mentagrophytes* strain. (**B**): The polymerase chain reaction results of *bar* (900 bp) gene amplification, the inserted *bar* gene was detected with primers *bar-*F and *bar*-R. Lane 1: *pDHt/ZafA-bar*, Lane 2: ZafA+bar, Lane 3: *pDHt/ZafA::hph*, Lane 4: ZafA-hph, Lane 5: The wild-type *T. mentagrophytes* strain. (**C**): The polymerase chain reaction results of *ZafA* gene (400 bp) amplification, the inserted *ZafA* gene was detected with primers *ZafA*q-F and *ZafA*q-R. Lane 1: The wild-type *T. mentagrophytes* strain, Lane 2: *pDHt/ZafA-bar*, Lane 3: ZafA+bar, Lane 4: *pDHt/ZafA::hph*, Lane 5: ZafA-hph. (**D**): The Southern blot results of *ZafA* gene, in which a partial *ZafA* gene fragment (304 bp) was used as a hybridization probe for detecting the deleted gene fragment. Lane 1: The wild-type *T. mentagrophytes* strain, Lane 2: *pDHt/ZafA::hph*, Lane 3: ZafA-hph, Lane M: DNA Molecular-Weight Marker, Lane 4: The wild-type *T. mentagrophytes* strain, Lane 5: *pDHt/ZafA-bar*, Lane 6: ZafA+bar, Lane 7: *pDHt/ZafA::hph*, Lane 8: ZafA-hph, Lane C: Positive fragments.

To confirm that *pDHt/ZafA::hph* and *pDHt/ZafA-bar* underwent homologous recombination in the recipient wild-type *T. mentagrophytes* strain and ZafA-hph cells, respectively, PCR and Southern blot analyses were performed. PCR indicated that the *hph* inserted into the *ZafA* locus in ZafA-hph, while *ZafA* and *bar* inserted into the *hph* open reading frame in ZafA+bar (Figure 1A–C). The Southern blot confirmed that *ZafA* was disrupted in ZafA-hph and restored in ZafA+bar (Figure 1D). These data indicated that the *ZafA* gene was successfully mutated in ZafA-hph and successfully restored in ZafA+bar.

### 2.2. Growth Abilities of the Wild-Type T. mentagrophytes Strain, ZafA-hph, and ZafA+bar

**Figure 2:**
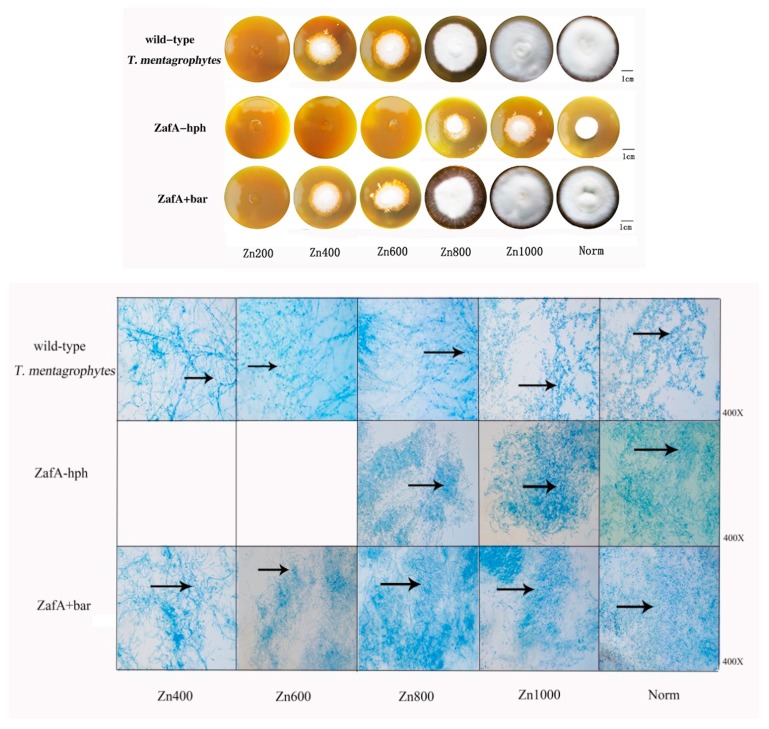
The growth ability of wild-type *T. mentagrophytes* strain, ZafA-hph, and ZafA+bar. Sabouraud dextrose agar (SDA) medium with one mM of N,N,N′,N′-tetrakis (2-pyridinylmethyl)-1,2-ethanediamine (TPEN) was supplemented to generate zinc deficient SDA medium, which was named SDA-Zn. The wild-type *T. mentagrophytes* strain, ZafA-hph, and ZafA+bar were inoculated to SDA medium (grouped into Norm) and SDA-Zn medium with 200 μM, 400 μM, 600 μM, 800 μM, and 1000 μM of zinc sulfate (grouped into Zn200, Zn400, Zn600, Zn800, and Zn1000) respectively. In SDA-Zn medium, there was no significant difference between the wild-type *T. mentagrophytes* strain and ZafA+bar in the growth performance (size of the colony), the number of spores, and the quality of mycelium. However, the growth performance (size of the colony) and reproduction ability of ZafA-hph were significantly lower than the wild-type *T. mentagrophytes* strain and ZafA+bar. The spores and mycelium are stained with lactate gossypol blue. The blue dots are spores, as shown at the black arrow. The denser of the blue dots, the more spores, and the more blue lines, the more mycelium.

The wild-type *T. mentagrophytes* strain, ZafA-hph, and ZafA+bar were inoculated into the zinc-deficient Sabouraud dextrose agar (SDA-Zn) medium and Sabouraud glucose liquid medium (zinc ions were chelated) with different zinc concentrations at 28 °C for 14 days. In the SDA-Zn medium, no significant differences were observed between the wild-type *T. mentagrophytes* strain and ZafA+bar in growth performance, spore number, or mycelial quality. However, ZafA-hph’s growth performance and reproduction ability were significantly lower than those of the wild-type *T. mentagrophytes* strain and ZafA+bar (Figure 2). 

**Figure 3:**
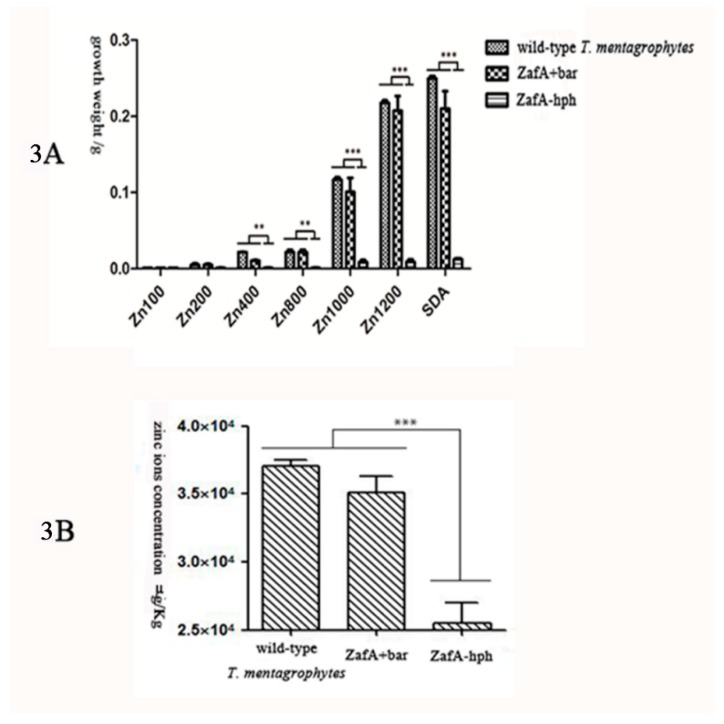
(**A**): The growth weight of wild-type *T. mentagrophytes* strain, ZafA-hph, and ZafA+bar. In Sabouraud glucose liquid medium, there was no significant growth weight difference in ZafA+bar and the wild-type *T. mentagrophytes* strain. However, the difference between ZafA-hph and the wild-type *T. mentagrophytes* strain was significant in growth weight. ** *p* < 0.05; *** *p* < 0.01. (**B**): The zinc absorption capacity of wild-type *T. mentagrophytes* strain, ZafA-hph, and ZafA+bar. The zinc ions concentration of the wild-type *T. mentagrophytes* strain, ZafA-hph, and ZafA+bar were determined by inductively coupled plasma-mass spectrometry (ICP-MS). Relative to the wild-type *T. mentagrophytes* strain and ZafA+bar, the zinc ions concentration was significantly lower in the ZafA-hph. ** *p* < 0.05; *** *p* < 0.01.

In the Sabouraud glucose liquid medium, no significant differences were observed in the growth weight of ZafA+bar or the wild-type *T. mentagrophytes* strain. However, ZafA-hph, the wild-type *T. mentagrophytes* strain, and ZafA+bar differed significantly in growth performance and weight (Figure 3A). These results show that the absence of the *ZafA* gene can significantly negatively affect *T. mentagrophytes*’ growth.

### 2.3. Zinc Absorption Capacities of the Wild-Type T. mentagrophytes Strain, ZafA-hph, and ZafA+bar

The zinc ion concentrations of the wild-type *T. mentagrophytes* strain, ZafA-hph, and ZafA+bar were determined by inductively coupled plasma-mass spectrometry (ICP-MS). Relative to the wild-type *T. mentagrophytes* strain and ZafA+bar, the zinc ion concentration was significantly lower in ZafA-hph (Figure 3B). These results show that the absence of *ZafA* can significantly negatively affect *T. mentagrophytes’* zinc absorption ability.

### 2.4. In Vitro Biodegradation of Hair

**Figure 4:**
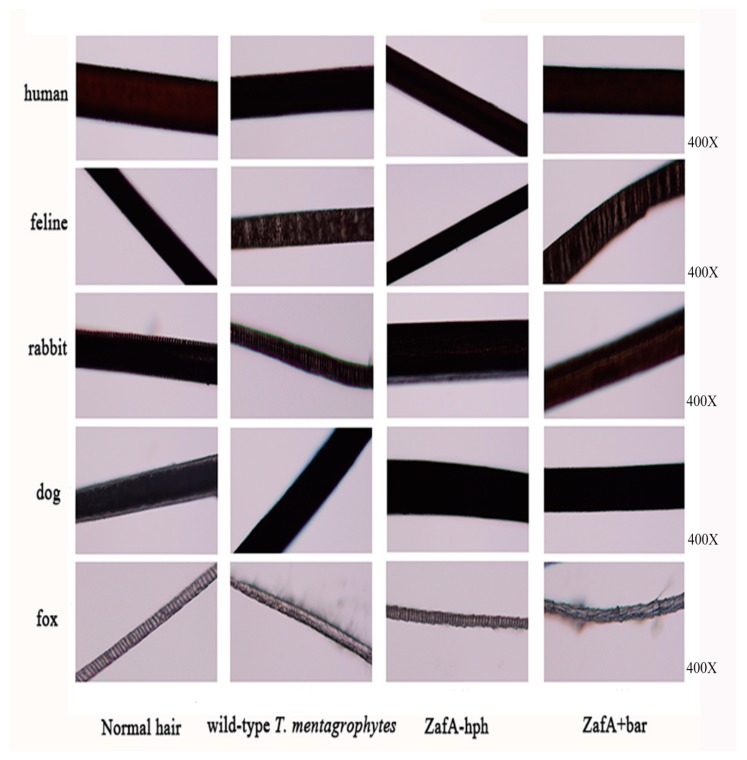
In vitro biodegradation of hair. The hair biodegradation test examined the susceptibility of different animal hair to the test strains of *T. mentagrophytes*. No obvious traces of pathological changes or decomposition were found in human and dog hair. The fox and feline hair were highly susceptible to *T. mentagrophytes*, and the rabbit hair was slightly susceptible to *T. mentagrophytes*. The decomposition significantly reduced on fox and cat’s hair in ZafA-hph, the pathological changes or decomposition on fox and cat’s hair have no obvious difference in ZafA+bar and the wild-type *T. mentagrophytes* strain.

The hair biodegradation test was used to examine the susceptibility of different animal hairs to the *T. mentagrophytes* test strains. The wild-type *T. mentagrophytes* strain, ZafA-hph, and ZafA+bar were co-cultured with different hair types in mineral culture medium. No obvious pathological changes or decomposition were found in the human or dog hair, suggesting low susceptibility to the *T. mentagrophytes* test isolates (Figure 4). In contrast, the fox and feline hair were highly susceptible to *T. mentagrophytes*, with obvious hair damage and perforation, and the rabbit hair was slightly susceptible to *T. mentagrophytes*. We found significantly reduced decomposition in the fox and cat hair with ZafA-hph, while the pathological changes and decomposition in the fox and cat hair did not obviously differ with the ZafA+bar or wild-type *T. mentagrophytes* strain. These results showed that *ZafA* plays an important role in hair biodegradation by *T. mentagrophytes* (Figure 4).

### 2.5. Animal Skin Inoculation Test

**Figure 5:**
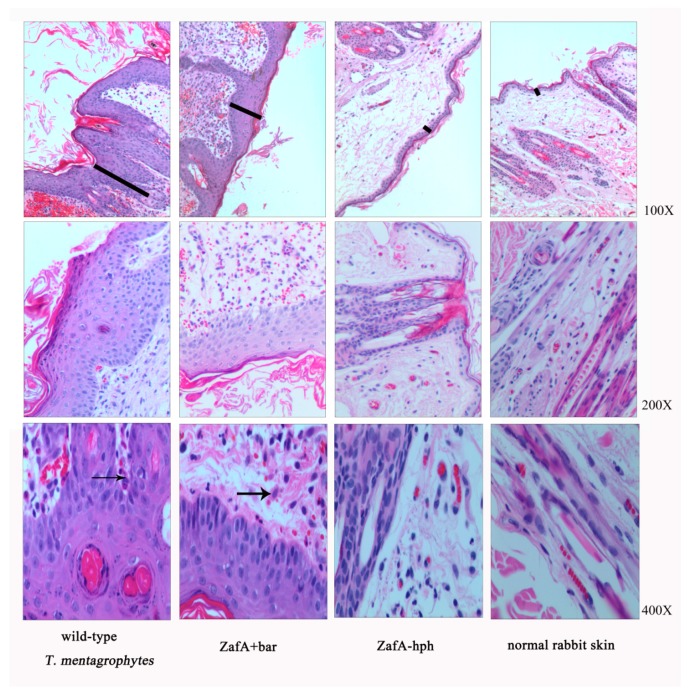
Animal skin inoculation test. Histopathological analysis of sections of skin infected with three strains. Sections were stained with hematoxylin–eosin (HE). Compared to normal rabbit skin, there were different degrees of thickening of the stratum corneum and the stratum spinosum layers (as shown by the black line), with an initial infiltration of inflammatory cells (as shown by the black arrow) following the inoculation of the wild-type *T. mentagrophytes* strain and ZafA+bar. However, the rabbit skin had a very slight lesion following inoculation with ZafA-hph, while there was no obvious difference with the normal skin.

The three strains were inoculated into rabbit skin to compare their pathogenic abilities, and the infection sites were observed each week following inoculation. Compared with normal rabbit skin, different degrees of thickening of the stratum corneum and stratum spinosum layers were observed, with initial inflammatory cell infiltration after inoculating the wild-type *T. mentagrophytes* strain and ZafA+bar (Figure 5). The rabbit skin did show very slight lesions after inoculation with ZafA-hph, but with no obvious differences from the normal skin (Figure 5). This indicates that the *ZafA* gene plays an important role in *T. mentagrophytes’* pathogenic ability.

## 3. Discussion

*T. mentagrophytes* is an important zoonotic skin ringworm pathogen that is distributed worldwide and can cause skin infections in humans and animals with serious threats to human and animal skin health. However, little research has been conducted on gene functioning in *T. mentagrophytes*. Therefore, deeper research is needed on *T. mentagrophytes*, which has great significance to public health.

Among fungal transformation techniques, *Agrobacterium tumefaciens*-mediated transformation (ATMT) has become a preferred method because of its high transformation efficiency and genetically stable transformants. At present, more than 180 binary vectors can be used for fungal ATMT, including *pCAMBIA*, *pGreen,* and *pCB301*, which have multiple cloning sites in the T-DNA region and are known as “empty T-DNA vectors”, allowing the easy insertion of selection markers, reporter genes, promoters, and terminators [29,30]. In this study, the plasmid *pDHt/SK* was obtained by inserting a 0.8-kb fragment from *pGreenII* into *pDHt* [22,31], which was constructed based on *pCAMBIA1300* by Mullins [32]. Through these modifications, plasmid *pDHt/SK* has more polyclonal sites, which facilitate inserting the exogenous gene. *Agrobacterium* EHA105 is a high-virulence strain that has been applied to many fungi for genetic transformation. In 2009, Yamada [27] finished the first genetic transformation of *T. mentagrophytes* through the EHA105 strain; then, Xi-ke Zhang and Yao Shi [28,33] used the EHA105 strain to successfully complete *T. mentagrophytes Mep1-5* and *Sub6* genetic transformations, respectively. Therefore, we chose *Agrobacterium* EHA105 for this study to finish the *T. mentagrophytes* genetic transformation, and we obtained the stable transformant, for which the highest transformation efficiency was 77 transformed strains/10^7^ conidia.

The researchers used homologous flank fragments of 600 bp, 800 bp, 1500 bp, and 2000 bp to finish the *T. mentagrophytes* homologous recombination. Li Zhang [34] used ATMT to knock out the *Brn1* gene in *Curvularia lunata*, and found that the homologous flank sequence length extended to more than 800 bp from 400 bp, and the *Brn1* gene’s homologous recombination rate could be increased from 60% to 100%. Homologous gene recombination efficiency is believed to depend mainly on the length of the homologous flank sequence on the vector, and the homologous recombination rate can be increased by increasing the size of the target gene’s homologous flank fragment. In this study, using the 1000-bp homologous flank sequences, the transformation efficiency was 55–77 transformed strains/10^7^ conidia, but only two transformed strains had *ZafA* gene homologous recombination in 50 transformed strains, which was lower than any other fungal homologous recombination rate [35]. This result may be related to differences in the specific gene locus, as the target gene locus can also affect the homologous recombination rate [36].

Zinc is an important micronutrient that is often combined with various functional proteins in fungi to form zinc finger proteins to complete biological functions. To maintain the proper zinc ion concentrations in fungal cells and ensure that fungal cells function normally, the complex zinc absorption and transport system has evolved [17]. At present, various fungal zinc absorption and transportation regulation factors have been described, and these proteins as zinc-responsive activity factors can regulate the expression of various zinc transporters at the transcription level, thus controlling the fungal zinc ion intake. Therefore, determining the function of zinc-responsive activity factors in *T. mentagrophytes* growth and pathogenicity studies is highly significant. In yeast, the zinc absorption and transport system is mainly regulated by the C_2_H_2_-type zinc finger transcription factor, Zap1 (zinc-responsive activity factor), which plays an important role in yeast growth [3]. Researchers found that various fungi can secrete C_2_H_2_ zinc finger transcription factors with similar functions. The main function of the *ZafA* protein in *A. fumigatus* is to adjust the zinc ion intake, and the *ZafA* gene is essential for growth when the zinc concentration is limited [18]. In *C. albicans*, Kim [19] first identified the *Csr1/Zap1* as a *Yeast Zap1* homologous gene, and also confirmed that the *Csr1/Zap1* mutant strain had a growth failure and could not form germ tubes or hyphae under zinc-deficient conditions. This indicates that *Csr1/Zap1* not only contributes to absorbing zinc ions, it also has an important effect on morphological transformations in *C. albicans*. Similar to other fungi, the zinc-responsive activity factor, Zap1, was also identified in *Cryptococcus*, and the *Zap1* gene mutant can decrease *Cryptococcal* growth ability under zinc-deficient conditions [20]. However, whether the zinc-responsive activity factor has similar functions in *T. mentagrophytes* and what its importance is to *T. mentagrophytes* remain unknown.

Therefore, in this study, we studied the function of the zinc-responsive activity factor in *T. mentagrophytes* using reverse genetics. In growth tests on the wild-type *T. mentagrophytes* strain, ZafA-hph, and ZafA+bar, we found that the hyphae quality, spore quantity, and growth weight of ZafA-hph were significantly reduced relative to the wild-type *T. mentagrophytes* strain, while ZafA+bar did not obviously differ from the wild-type *T. mentagrophytes* strain. Thus, the significant decrease in *T. mentagrophytes’* growth ability was due to the lack of the zinc-responsive activity factor, rather than the transformation process. The zinc-responsive activity factor of *T. mentagrophytes* was essential for its growth and reproduction. In detecting *T. mentagrophytes’* zinc absorption capacity, the intracellular zinc ion content of ZafA-hph was significantly decreased relative to the wild-type *T. mentagrophytes* strain, and no obvious differences were observed between the wild-type *T. mentagrophytes* strain and ZafA+bar. Thus, the zinc-responsive activity factor plays an important role in absorbing zinc ions in *T. mentagrophytes*.

The zinc-responsive activity factor not only depletes fungal growth capacity, it also affects its virulence to the host. The *A. fumigatus* mutant strain without the *ZafA* gene does not survive or cause disease in mice, indicating that the *ZafA* gene is necessary for *A. fumigatus* virulence [18]. In *C. gattii*, the *Zap1* gene also significantly affected virulence [20]. In this study, we conducted hair biodegradation and skin testing on *T. mentagrophytes* to study the zinc-responsive activity factor’s effect on *T. mentagrophytes* virulence. In the hair biodegradation test, human and dog hair were not sensitive to *T. mentagrophytes*, but *T. mentagrophytes* caused severe perforations in the cat and fox hair, which was consistent with the research of Zhang Xinke et al. [28]. In the biodegradation test on the cat and fox hair, *T. mentagrophytes’* hair biodegradation capacity nearly disappeared after the zinc-responsive activity factor was knocked out. However, when the gene was restored by the same gene transformation method, its hair biodegradation capacity did not significantly differ from that of the wild-type *T. mentagrophytes* strain. This indicates that the degradation ability of the hair biodegradation capacity is due to the lack of the zinc-responsive activity factor. In the animal skin inoculation experiment, we used rabbits as subjects, and inoculated the three strains into rabbit skin for 14 days, and then observed the tissue sections. The wild-type *T. mentagrophytes* strain and ZafA+bar resulted in significantly thickened cuticles and inflammatory cell infiltration in the rabbits. However, in ZafA-hph, the skin lesions nearly disappeared, and did not significantly differ from normal rabbit skin. These results showed that the zinc-responsive activity factor of *T. mentagrophytes* was important to host pathogenicity.

Thus, the *T. mentagrophytes* zinc-responsive activity factor plays an important role in growth, reproduction, and virulence. Studying its infection mechanisms is valuable for determining new drug targets and developing effective vaccines for *T. mentagrophytes*. Studying *T. mentagrophytes* is valuable and significant for effectively preventing and controlling *T. mentagrophytes* infections.

## 4. Materials and Methods

### 4.1. Strains and Media

The wild-type *T. mentagrophytes* strain, ATCC 28,185 (a gift from Ruoyu Li, Peking University First Hospital, Beijing, China), was cultured on solid Sabouraud dextrose agar medium (SDA) supplemented with 50 mg/mL chloramphenicol (Sigma, St. Louis, MO 63103, USA) at 28 °C [33]. The *T. mentagrophytes ZafA* mutant was selected on solid SDA medium supplemented with 600 μg/mL of hygromycin B (Sigma, St. Louis, MO 63103, USA) and 200 μg/mL of cephalexin (Sigma, St. Louis, MO 63103, USA). The *T. mentagrophytes ZafA complemented strain* was selected on solid SDA medium supplemented with 2 mg/mL of glufosinate-ammonium (Sigma, St. Louis, MO 63103, USA) and 200 μg/ml of cephalexin (Sigma, St. Louis, MO 63103, USA).

*Agrobacterium tumefaciens* strain EHA105 (a gift from Dr K. J. Kwon-Chung, National Institutes of Health, Bethesda, MD, USA) was maintained at 28 °C on solid yeast extract broth medium (YEB) supplemented with 50 μg/mL of rifampicin (Sigma, St. Louis, MO 63103, USA) and 25 μg/mL of chloramphenicol (Sigma, St. Louis, MO 63103, USA) [27]. An induction medium (AIM) was used for culturing *A. tumefaciens* carrying the binary vectors, *pDHt/ZafA::hph* or *pDHt/ZafA-bar*, supplemented with 50 μg/mL of rifampicin (Sigma, St. Louis, MO 63103, USA), 25 μg/mL of chloramphenicol (Sigma, St. Louis, MO 63103, USA), 50 μg/mL of kanamycin (Sigma, USA), and 200 μM of acetosyringone (Sigma, St. Louis, MO 63103, USA) [24,37].

### 4.2. Constructing the Transformation Vectors

**Figure 6:**
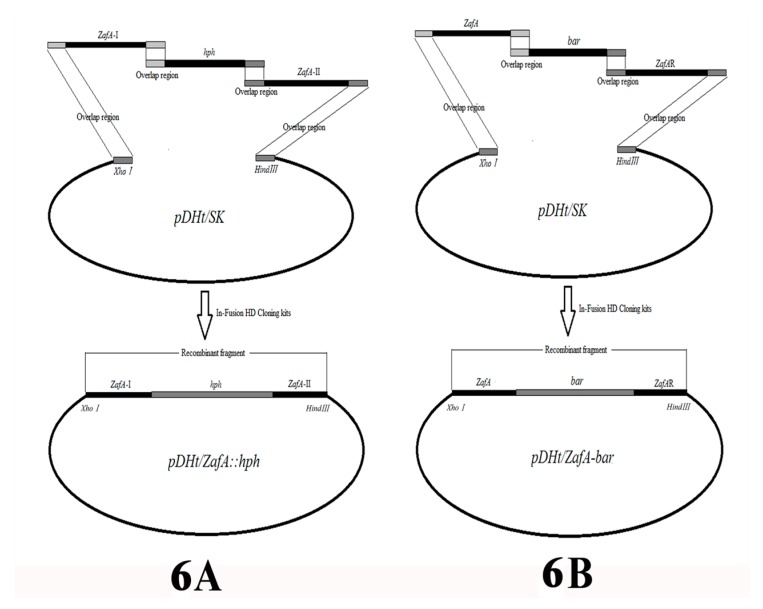
Construction of the transformation vectors. (**A**): The *hph* gene of *pAN7–1* and two flanking sequences of the *ZafA* gene were inserted simultaneously into plasmid *pDHt/SK* and digested by *XhoI/HindIII* by means of In-Fusion HD Cloning kits. The forward primer of *ZafA-I* has a 20-nt overlap region with the *pDHt/SK* digested by *XhoI*, and the reverse primer of *ZafA-I* has a 15-nt overlap region with the *hph*. The forward primer of *hph* has a 15-nt overlap region with the *ZafA-I* and the reverse primer of *hph* has a 15-nt overlap region with *ZafA-II*. The forward primer of *ZafA-II* has a 15-nt overlap region with the *hph* and the reverse primer of *ZafA-II* has a 20-nt overlap region with *pDHt/SK* digested by *HindIII*. (**B**): The *bar* gene of *pBARGPE1-mCherry*, right flanking sequences of the *ZafA* gene, and whole *ZafA* gene were inserted simultaneously into plasmid *pDHt/SK* and digested by *XhoI/HindIII* by means of In-Fusion HD Cloning kits. The forward primer of *ZafA* has a 20-nt overlap region with the *pDHt/SK* digested by *XhoI* and the reverse primer of *ZafA* has a 15-nt overlap region with the *bar*. The forward primer of *bar* has a 15-nt overlap region with the *ZafA*, and the reverse primer of *bar* has a 15-nt overlap region with *ZafAR*. The forward primer of *ZafAR* has a 15-nt overlap region with the *bar* and the reverse primer of *ZafAR* has a 20-nt overlap region with *pDHt/SK* digested by *HindIII*.

The binary vector, *pDHt/ZafA::hph*, which is used for insertional mutagenesis was constructed by fusing the hygromycin B resistance gene (*hph*) and the left and right flanking sequences of the *ZafA* gene of *T. mentagrophytes*. They were inserted simultaneously into the *pDHt/SK* plasmid (a gift from Dr. K. J. Kwon-Chung) digested by *XhoI/HindIII* using In-Fusion HD cloning kits (Takara, Nojihigashi 7-4-38, Kusatsu, Shiga 525-0058, Japan) in strict accordance with the instruction manual (Figure 6A) [38,39]. The *hph* gene was obtained by PCR using primer pairs, *HphF* and *HphR*, from plasmid *pAN7-1* (overlap sequence inserted by PCR amplification; see Table 1 for primer sequences). The left and right flanking fragments (*ZafA*-I nucleotide position: −192 to 735; *ZafA*-II nucleotide position: 1603 to 2565) of the *ZafA* gene were amplified using the primer pairs, *ZafA*I-F/*ZafA*I-R and *ZafA*II-F/*ZafA*II-R, respectively (overlap sequence inserted by PCR amplification; see Table 1 for primer sequences). The binary vector *pDHt/ZafA::hph* was transformed into *A. tumefaciens* strain EHA105 using the heat-shock method. All of the fragments that were amplified or inserted into the plasmid were verified by DNA sequencing.

The binary vector *pDHt/ZafA-bar* that was used for complemented *ZafA* was constructed by fusing the glufosinate-ammonium resistance gene (*bar*) and the complete *ZafA* gene and right flanking sequences of the *ZafA* gene of *T. mentagrophytes*, which were inserted simultaneously into plasmid *pDHt/SK* digested by *XhoI/HindIII* using In-Fusion HD cloning kits (Takara, Nojihigashi 7-4-38, Kusatsu, Shiga 525-0058, Japan) in strict accordance with the instruction manual (Figure 6B). The *bar* gene was obtained by PCR using primer pairs, *BarF* and *BarR*, from plasmid *pBARGPE1-mCherry* (Wuhan Jingxiu Scientific Research Biotechnology Co. Ltd., China) (overlap sequence inserted by PCR amplification; see Table 1 for primer sequences). The complete *ZafA* gene and right flanking fragments of the *ZafA* gene were amplified using primer pairs, *ZafA*-F/*ZafA*-R and *ZafA*R-F/*ZafA*R-R, respectively (overlap sequence inserted by PCR amplification; see Table 1 for primer sequences). The binary vector *pDHt/ZafA-bar* was transformed into the *A. tumefaciens* strain EHA105 using the heat-shock method. All of the fragments that were amplified or inserted into the plasmid were verified by DNA sequencing.

### 4.3. ATMT Transformation

*A. tumefaciens* strain EHA105 transformants carrying *pDHt/ZafA::hph* or *pDHt/ZafA-bar* were grown on solid YEB medium supplemented with 50 μg/mL of rifampicin, 25 μg/mL of chloramphenicol, and 50 μg/mL of kanamycin at 28 °C for 48 h, collected in 10 mL of sterile 0.9% sodium chloride solution, centrifuged at 3500 rpm/min, collected in 10 mL of the liquid YEB medium (50 μg/mL kanamycin, 50 μg/mL rifamycin, and 25 μg/mL chloramphenicol), and cultured for 48 h at 28 °C. After centrifuging at 3500 rpm/min, the *A. tumefaciens* cells were suspended in liquid AIM to an optical density of 0.7 at 660 nm. After adding 200 μM of acetosyringone, the bacterial suspensions were incubated on a shaker at 150 rpm/min at 28 °C for 6 h. 

The wild-type *T. mentagrophytes* strain 28,185 was cultured on solid SDA medium at 28 °C for 14 days, then gently washed with sterile 0.9% sodium chloride solution, and the conidial concentration was adjusted to 1 × 10^7^ colony-forming units (CFU)/mL. A mixture of 100 μL of *A. tumefaciens* suspension and 100 μL of *T. mentagrophytes* conidial suspension was spread onto sterilized nylon membranes (Sigma, USA), placed on solid AIM medium (supplemented with 200 μM of acetosyringone) and incubated at 28 °C in the dark for 60 h. The nylon membranes were transferred onto a solid SDA medium plate supplemented with 600 μg/mL of hygromycin B and 50 μg/mL of cephalexin, and incubated at 28 °C for two to three days [28,40]. After two to three days, fungal colonies were produced on the nylon membranes, and 10 samples were randomly chosen on one solid SDA medium plate and cultivated on solid SDA medium supplemented with 600 μg/mL of hygromycin B at 28 °C for 14 days. The *T. mentagrophytes ZafA* mutant was named ZafA-hph. The *T. mentagrophytes ZafA* complemented strain was obtained by the same methods as above, then selected by 2 mg/mL of glufosinate-ammonium and named ZafA+bar.

### 4.4. Total DNA Isolation and Hybridization Analysis

ZafA+hph and ZafA+bar were analyzed by polymerase chain reaction (PCR) and Southern blot analyses to confirm the disruption and restoration of the *ZafA* gene. Total DNA from the wild-type *T. mentagrophytes* strain, ZafA+hph, and ZafA+bar were extracted from growing mycelia using sterile acid-washed glass beads (Sigma, USA), as previously described [41]. The inserted *hph* and *bar* genes were detected using primers *hph*-F and *hph*-R and *bar-*F and *bar*-R (see Table 1 for primer sequences), respectively. Specific gene disruption and restoration were confirmed using primers *ZafA*q-F and *ZafA*q-R (see Table 1 for primer sequences).

Southern blot hybridization analysis was used to determine whether the mutant and restoration strains showed successful homologous recombination. The hybridization probe was used to detect the *ZafA* fragment that should be replaced by *hph* if the mutant was successful in ZafA-hph, and the *ZafA* gene that should be recovered if the restoration was successful in ZafA+bar (probe-1, 304 bp; see Table 1 for primer sequences).

Total DNA was digested by *XhoI*, resulting in target fragments. Digested samples were separated by electrophoresis on 1% (*w/v*) agarose gels and transferred onto Hybond N+ membranes (Pharmacia, Piscataway, NJ, USA). Probes were labeled with digoxigenin-dUTP using the DIGD-110 Labeling Kit (Roche, Basel, Switzerland). Hybridization and signal detection were conducted per the manufacturer’s instructions.

### 4.5. Determining Growth Ability

SDA medium with one mM of N,N,N′,N′-tetrakis (2-pyridinylmethyl)-1,2-ethanediamine (TPEN) was used to generate the zinc-deficient SDA medium, SDA-Zn (zinc ions were chelated). The wild-type *T. mentagrophytes* strain, ZafA-hph, and ZafA+bar, were inoculated into the SDA medium (sufficient zinc ions, grouped into Norm) and SDA-Zn medium with 200 μM, 400 μM, 600 μM, 800 μM, and 1000 μM of zinc sulfate (grouped into Zn200, Zn400, Zn600, Zn800, and Zn1000, respectively). The culture conditions were 28 °C for 14 days to observe the fungal growth. The same culture methods were performed in the Sabouraud glucose liquid medium, and the centrifuged fungi were weighed.

### 4.6. Determining Zinc Absorption Capacity

The wild-type *T. mentagrophytes* strain, ZafA-hph, and ZafA+bar, were maintained at 28 °C on SDA medium for 14 days. Sterile 0.9% sodium chloride solution (5 mL) was used to wash off the spores to collect the fungal liquid. The fungal liquid concentration was adjusted to 10^8^ CFU/mL using cell count plates. The 100-μL fungal liquid was inoculated onto sterilized nylon membranes (Sigma, USA) and placed on solid SDA medium at 28 °C for 14 days. Next, 0.2 g of the fungus was scraped from the nylon membrane surface, and the zinc concentration in the fungi was determined by inductively coupled plasma-mass spectrometry (ICP-MS).

### 4.7. In Vitro Biodegradation of Hair

Healthy human, dog, cat, rabbit and fox hair were cut into one-centimeter pieces, sterilized, and used as substrates for biodegradation assays [42]. All of the experimental methods, operations, and protocols were approved by the animal ethics committee of Northwest Agriculture and Forest University. Conidial suspensions (200 μL, 10^8^ CFU/mL) of the wild-type *T. mentagrophytes* strain, ZafA-hph, and ZafA+bar were added to 10 mL of mineral medium to assess the fungal biodegradation capability of the five hair types (100 mg) at 28 °C for 28 days [43]. The degree of hair degradation was recorded.

### 4.8. Animal Inoculation Test

A total of 25 adult white rabbits consisting of 10 males and 15 females and weighing between 2–3 kg were selected for the inoculation test. All of the adult white rabbits were purchased from Northwest A & F University Animal Laboratories (Xian, China). The animals were housed for seven days prior to testing. All of the rabbits were reared, obtained, and housed in accordance with our institute’s laboratory animal requirements, and all of the animals’ procedures and study design were conducted in accordance with the Guide for the Care and Use of Laboratory Animals (Ministry of Science and Technology of China, 2006) and were approved by the animal ethics committee of Northwest A & F University (20170621-2). The rabbits were randomly divided into the wild-type *T. mentagrophytes* strain (*n* = 6), ZafA-hph (*n* = 6), ZafA+bar (*n* = 6), and negative control (*n* = 3) groups. An area of 2.5 × 2.5 cm^2^ on each animal’s abdomen was shaved using an electric shaver one day prior to inoculation. Following routine sterilization procedures, 200 μL (1 × 10^8^ CFU) conidial suspensions of the wild-type *T. mentagrophytes* strain, ZafA-hph, and ZafA+bar were inoculated separately into the skin within the shaved abdominal area, and sterile 0.9% sodium chloride solution was inoculated into the control animals. All of the rabbits were housed in separate cages to avoid contamination, and clinical symptoms were observed and recorded daily. After 14 days, three rabbits were randomly selected from each test group, one animal was randomly selected from the control group, and 8.5-mm diameter skin biopsies were taken surgically. Skin infections were evaluated histologically.

### 4.9. Statistical Analyses

Assays had been repeated three times; one-way analysis of variance (ANOVA) was used for the statistical comparisons between different groups. The tests were performed using IBM SPSS Statistics 24 software (SPSS Inc., Chicago, IL, USA). *p* < 0.05 was considered to be statistically significant.

## Figures and Tables

**Table 1:** The primers sequences.

Name	Sequence (5′–3′)
*HphF*	CTTAATCACCTTCACAAGCGAAGGAGAATGTGAAGCC
*HphR*	TTAGTGACGAGCAGCGCTGTATCTGGAAGAGGTAAAC
*ZafA*I-F	GTACCGGGCCCCCCCTCGAGTATCTGCGAGACACTGGACGAT
*ZafA*I-R	CATTCTCCTTCGCTTGTGAAGGTGATTAAGGTAAGGG
*ZafA*II-F	TCTTCCAGATACAGCGCTGCTCGTCACTAACATTGTT
*ZafA*II-R	AGGAATTCGATATCAAGCTTGGAGATAGAGAATGCGGTTAAA
*BarF*	GCATTCTCTATCTCCCTCATCAGATAACAGCAATACC
*BarR*	TTAGTGACGAGCAGCCGCCACATAGCAGAACTTTAAA
*ZafA*-F	GTACCGGGCCCCCCCTCGAGTATCTGCGAGACACTGGACGAT
*ZafA*-R	CTGTTATCTGATGAGGGAGATAGAGAATGCGGTTAAA
*ZafA*R-F	TTCTGCTATGTGGCGGCTGCTCGTCACTAACATTGTT
*ZafA*R-R	AGGAATTCGATATCAAGCTTGGAGATAGAGAATGCGGTTAAA
*hph*-F	TACATCCATACTCCATCCTTC
*hph*-R	CGGCATCTACTCTATTCCTT
*bar-*F	AGTTATTAGGTCTGAAGAGGAG
*bar*-R	CCATCGTCAACCACTACAT
*ZafA*q-F	CCAGACTGAAGGTGCTAAG
*ZafA*q-R	CCTGTTAGTATCGTCGTGTT
*probe-1F*	GCTCCATCCTTTCATTCG
*probe-1R*	TTCCCTTAGCACCTTCAGT

Underline base is the overlap region.
